# Resilience factors may buffer cellular aging in individuals with and without chronic knee pain

**DOI:** 10.1177/1744806919842962

**Published:** 2019-04-25

**Authors:** Alisa J Johnson, Ellen Terry, Emily J Bartley, Cynthia Garvan, Yenisel Cruz-Almeida, Burel Goodin, Toni L Glover, Roland Staud, Laurence A Bradley, Roger B Fillingim, Kimberly T Sibille

**Affiliations:** 1 Pain Research and Intervention Center of Excellence, University of Florida, Gainesville, FL, USA; 2 Department of Community Dentistry and Behavioral Science, College of Dentistry, University of Florida, Gainesville, FL, USA; 3 Department of Anesthesiology, University of Florida, Gainesville, FL, USA; 4 Department of Aging and Geriatric Research, Institute on Aging, University of Florida, Gainesville, FL, USA; 5 Division of Pain Medicine, Department of Anesthesiology and Perioperative Medicine, Department of Psychology, University of Alabama, Tuscaloosa, AL, USA; 6 College of Nursing, University of Florida, Gainesville, FL, USA; 7 Department of Medicine, University of Florida, Gainesville, FL, USA; 8 Division of Clinical Immunology and Rheumatology, Department of Medicine, University of Alabama at Birmingham, Tuscaloosa, AL, USA

**Keywords:** Resilience, telomeres, chronic pain, stress, osteoarthritis

## Abstract

Telomere length, a measure of cellular aging, is inversely associated with chronic pain severity. While psychological resilience factors (e.g., optimism, acceptance, positive affect, and active coping) are associated with lower levels of clinical pain and greater physical functioning, it is unknown whether resilience may buffer against telomere shortening in individuals with chronic pain. Additionally, a broader conceptualization of resilience that includes social and biobehavioral factors may improve our understanding of the relationship between resilience, chronic pain, and health outcomes. In individuals with and without chronic knee pain, we investigated whether (1) psychological resilience would be positively associated with telomere length and if (2) a broader conceptualization of resilience including social and biobehavioral factors would strengthen the association. Seventy-nine adults, 45 to 85 years of age, with and without knee pain completed demographic, health, clinical pain, psychological, social, and biobehavioral questionnaires. Resilience levels were determined by summing the total number of measures indicating resilience based on published clinical ranges and norms. Blood samples were collected, and telomere length was determined. In regression analyses controlling for sex, race, age, and characteristic pain intensity, greater psychological resilience and psychosocial/biobehavioral resilience were associated with longer telomeres (*p* = .0295 and *p* = .0116, respectively). When compared, psychosocial/biobehavioral resilience was significantly more predictive of telomere length than psychological resilience (*p* < .0001). Findings are promising and encourage further investigations to enhance understanding of the biological interface of psychosocial and biobehavioral resilience factors in individuals with musculoskeletal chronic pain conditions.

## Introduction

Telomere length (TL) is a measure of cellular aging and a downstream indicator of stress system functioning.^1,2^ Shorter telomeres are associated with maladaptive health behaviors, obesity, and increased morbidity and mortality.^3–5^ In contrast, longer telomeres are associated with positive psychological factors, exercise, and health behaviors.^6–9^ Further, clinical intervention studies indicate TL is influenced by positive health interventions.^10–12^ Thus, telomeres appear to serve as a biological indicator of the cumulative life experience: biological, psychosocial, and behavioral.^13^ Importantly, a number of studies have reported an inverse relationship between TL and the biological and psychosocial stress experienced with persisting chronic pain. For example, in individuals reporting high stress and chronic pain, TL was shorter compared to those individuals with low stress and no chronic pain.^14^ Similarly, TL was shorter among individuals with fibromyalgia reporting higher pain and comorbid depression compared to those with low levels of pain and depression.^15^ More recently, in a larger cohort of individuals with and without chronic knee pain, TL was associated with chronic pain severity in a dose-response fashion.^16^ TL may also reflect the buffering effect of resilience factors in individuals with chronic pain.

An array of studies indicates the clinical benefit of several psychological factors in the experience of chronic pain. Qualities such as positive affect, dispositional optimism, active coping, acceptance, and purpose in life have been indicated as buffering against the negative sequelae of chronic pain.^17–20^ These psychological factors are frequently described as promoting “resilience” as a result of their being associated with lower clinical pain and greater physical function.^20,21^ In essence, resilience is “the process by which people bounce back from adversity and reintegrate and ideally grow from the experience.”^22^ Hence, these psychological factors appear to be protective, limiting the negative impact of living with chronic pain.

In addition to psychological factors, social factors may also promote resilience in individuals with chronic pain.^20,23^ Indeed, social support and social integration predict lower all-cause mortality rates in various populations.^24–27^ Additionally, positive social experiences and higher levels of self-reported social integration were associated with lower biological “wear and tear” recognized as allostatic load (AL).^28^ Further, there is evidence that emotional and social support may attenuate biological responses to environmental stress.^29,30^

Resilience factors are typically regarded as psychological or psychosocial constructs.^19,20,31–34^ However, it may be more optimal to consider resilience from a more comprehensive biopsychosocial framework. For example, health behaviors such as regular exercise, moderate alcohol consumption, and being a non-smoker predict decreased morbidity and mortality in individuals with chronic widespread pain^35^ and have been linked with lower levels of inflammatory and metabolic biomarkers.^36^ Additionally, in individuals with or at risk for knee osteoarthritis (OA), lower omega-6:omega-3 polyunsaturated fatty acid (PUFA) ratios were associated with lower clinical pain, experimental pain sensitivity, and psychosocial distress, and greater physical functioning compared to those individuals with higher omega-6:omega-3 (O6:O3) PUFA ratio levels.^37^ Low O6:O3 PUFA ratios have also been associated with increased TL in overweight sedentary adults and may serve to enhance overall resilience.^10^ Thus, a broad conceptualization of resilience that includes psychosocial and biobehavioral resilience factors may be more informative in understanding the biological interface of resilience and chronic pain.

The aims of the current study were to determine (1) if greater levels of psychological resilience are associated with TL and (2) if a broader conceptualization of resilience that includes social and biobehavioral factors would improve the model. We hypothesized that (1) greater levels of psychological resilience and psychosocial/biobehavioral resilience would be associated with longer TL and that (2) greater levels of psychosocial/biobehavioral resilience would demonstrate stronger association with TL compared to psychological resilience.

## Methods

### Study design

The current investigation is a sub-study of a larger study, *Understanding Pain and Limitations in Osteoarthritic Disease* (UPLOAD). UPLOAD was a multi-site cross-sectional investigation conducted at the University of Florida (UF) and the University of Alabama at Birmingham (UAB). The study is limited to data collected at the University of Florida. All procedures were reviewed and approved by the UF Institutional Review Board (IRB201500906). Participants provided written informed consent. The Methods section is limited to the components applicable to the current study. A complete description of the overall study is available.^38^

### Participants

Participants in the current investigation were enrolled at the University of Florida during the period between 2010 and 2013, and had TL measures completed. The sample consisted of 79 community-dwelling adults between 45 and 85 years of age, with and without knee pain in the previous month, who self-identified as non-Hispanic Black (NHB) or non-Hispanic White (NHW). Individuals were excluded from the study for the following self-reported conditions: (1) prosthetic knee replacement or other clinically significant surgery to the painful knee; (2) uncontrolled hypertension (blood pressure > 150/95 mm Hg); (3) heart disease, congestive heart failure, or history of acute myocardial infarction; (4) severe peripheral neuropathy in which pain testing was contraindicated; (5) systemic rheumatic disorders including rheumatoid arthritis, systemic lupus erythematosus, gout, and fibromyalgia; (6) neurological diseases such as Parkinson’s disease, multiple sclerosis, stroke with loss of sensory or motor function, or uncontrolled seizures; (7) significantly greater pain in other body sites than in the knee; (8) chronic daily opioid use; (9) hospitalization within the preceding year for psychiatric illness; or (10) pregnant or nursing.

### Procedures

Participants completed a standardized telephone screening to confirm initial eligibility. Eligible participants completed two sessions approximately two to three weeks apart. During Session 1, after written informed consent was obtained, participants completed a comprehensive health assessment, which involved the collection of demographic information, anthropometric measurements, health history information, and physical examination. During Session 2, blood samples were collected, and peripheral blood mononuclear cells were isolated to assess TL. Participants completed a number of self-report questionnaires to assess clinical pain, psychological, social, and behavioral factors.

### Measures

#### Chronic pain

The Graded Chronic Pain Scale (GSPC) is a self-report questionnaire that assesses chronic pain intensity and pain-related disability.^39^ Characteristic pain intensity is determined by a response to three questions on a 0 to 10 numeric rating scale: current, worst, and average knee pain intensity during the past six months. Ratings from the three items are averaged and multiplied by 10 to calculate a score (0–100).^39^ The Graded Chronic Pain Scale (GCPS) has demonstrated internal consistency (Cronbach’s α’s = 0.74).^39^ Characteristic pain intensity was used as a covariate in the analyses.

#### Psychological resilience

Validated and recognized measures were used to operationalize the psychological resilience phenotype. For each measure, if the score fell within the positive/protective range based on clinical/normative values a score of 1 was added for resilience. A total psychological resilience value was determined based on the summative total from the combined measures.

##### Dispositional optimism

The Life Orientation Test-Revised (LOT-R) is a 10-item questionnaire that assesses dispositional optimism and pessimism. The scale contains three items for optimism (e.g., “In uncertain times, I usually expect the best”) and pessimism (e.g., “If something can go wrong for me, it will”), with four non-contributing questions. Responses are based on a four-point Likert-type scale (0 = “strongly disagree” to 4 = “strongly agree”).^40^ The measure has demonstrated a test–retest reliability of .79 at 28 months and Cronbach’s alpha of .82.^40^ Response totals ≥18 indicated resilience.^40^

##### Positive and negative affect

Positive and negative affect were assessed using the Positive and Negative Affect Scale (PANAS).^41,42^ The PANAS is a 20-item measure that consists of 10 positively valenced items (positive affect) and 10 negatively valenced items (negative affect). Items are self-rated on a five-point Likert-type scale (1 = “very slightly or not at all” to 5 = “extremely”) and summed to produce total scores for each affect component, with higher scores representing higher levels of the construct. The timeframe collected in this study was “to what extent you generally feel this way.” The PANAS has been demonstrated to be internally consistent (Cronbach’s α’s ≥ .84).^41,42^ Having high levels of trait positive affect and low levels of trait negative affect are considered as positive and resilience promoting.^43–46^ Response totals for positive affect ≥35 and negative affect ≤18.1 indicated resilience.^41,45^

##### Active coping

Active coping was assessed using the Coping Strategies Questionnaire-Revised (CSQ-R), a 27-item measure of pain coping strategies.^47–49^ Participants rate how often they use each strategy on a 0 (“never do that”) to 6 (“always do that”) Likert scale. Items are summed for each domain separately with higher scores indicating greater use of that strategy.^50^ The CSQ-R has demonstrated acceptable reliability (Cronbach’s α’s = 0.72–0.86).^49,51^ Responses on the CSQ-R have been associated with arthritis pain and disability in both NHB and NHW individuals.^52^ Active coping was assessed using items from the distraction, ignoring pain sensations, distracting from pain, and coping self-statements domains. Based on prior findings, response totals >2.87 indicated resilience.^47^

##### Perceived stress

Perceived stress was assessed through the 10-item Perceived Stress Scale (PSS), a reliable (Cronbach’s α’s ≥ 0.84) and valid scale designed to measure the role of non-specific appraised stress.^53,54^ Participants are asked to rate (0 = “never” to 4 = “very often”) statements asking about thoughts and feelings over the past month. A total perceived stress score is computed. Low levels of perceived stress are considered as a positive psychological factor and have been associated with TL.^2,8,14^ Based on recommended ranges, perceived stress scores ranging from 0 to 13 indicated resileince.^53–55^

#### Pbiobehav/biobehavioral resilience

In addition to the psychological measures described above, three additional validated and recognized measures were included to assess a psychosocial and biobehavioral conceptualization of resilience.

##### Social support

The Multidimensional Scale of Perceived Social Support (MSPSS) is a 12-item self-report measure used to assess the extent to which an individual perceives social support from family, friends, and significant others.^56^ Each item is rated on a seven-point Likert scale (1 = “very strongly disagree” to 7 = “very strongly agree”), for a total score ranging from 12 to 84, with higher scores indicating higher levels of perceived support. The MSPSS has been demonstrated to be a reliable measure across subscales (Cronbach’s α’s = 0.81—0.98).^57,58^ Based on published ranges, scores in the range of 49 to 84 indicated resilience.^56^

##### Tobacco use

Participants responded to a question regarding smoking status: never, former, or current. Responses endorsing never and former smoker status indicated resilience.

##### Waist-to-hip ratio

Participant’s waist circumference and hip circumference were measured using a measuring tape. The waist-to-hip ratio (WHR) is determined by dividing the wait circumference by the hip circumference. The World Health Organization defines abdominal obesity as a WHR of > .85 for women and > .90 for men.^59^ Risk and protective ranges were determined applying a generous approach. A WHR < .90 for both men and women was defined as indicating resilience.

### TL analysis

TL analysis followed a standardized procedure as previously described.^16^

A blood sample was collected during the second study session and placed on ice. It was centrifuged at 4°C for 10 min at 3000 r/min. The blood was mixed with 1× phosphate-buffered saline, layered onto a volume of Lymphoprep solution that was contained in a centrifuge tube. After centrifugation, the lymphocyte band was separated, washed, and centrifuged to form a pellet. The pellet was resuspended in 1× phosphate-buffered saline, and the sample is stored at –80°C. The DNA isolation was achieved using the Qiagen Flexigene kit. Lysis buffer was added to the sample before being mixed and centrifuged. The resulting pellet was resuspended in denaturation buffer containing protease and incubated. DNA was then precipitated, washed, centrifuged, and resuspended in hydration buffer. TL was analyzed by the Blackburn Lab, University of California San Francisco.^60^

The TL assay is adapted from the published original method by Cawthon.^60,61^ The telomere thermal cycling profile consists of Cycling for T(telomic) polymerase chain reaction (PCR): 96°C for 1 min; denature at 96°C for 1 s, anneal/extend at 54°C for 60 s, with fluorescence data collection, 30 cycles. Cycling for S (single copy gene) PCR: PCR: 96°C for 1 min; denature at 95°C for 15 s, anneal at 58°C for 1 s, extend at 72°C for 20 s, 8 cycles; followed by denature at 96°C for 1 s, anneal at 58°C for 1 s, extend at 72°C for 20 s, hold at 83°C for 5 s with data collection, 35 cycles. The primers for the telomere PCR are tel1b [5′-CGGTTT(GTTTGG)_5_GTT-3′], used at a final concentration of 100 nM, and tel2b [5′-GGCTTG(CCTTAC)_5_CCT-3′], used at a final concentration of 900 nM. The primers for the single-copy gene (human beta-globin) PCR are hbg1 [5′-GCTTCTGACACAACTGTGTTCACTAGC-3′], used at a final concentration of 300 nM, and hbg2 [5′-CACCAACTTCATCCACGTTCACC-3′], used at a final concentration of 700 nM. The final reaction mix contains 20 mM Tris-HCl, pH 8.4; 50 mM KCl; 200 mM each dNTP; 1% DMSO; 0.4× Syber Green I; 22 ng E.coli DNA per reaction; 0.4 Units of Platinum Taq DNA polymerase (Invitrogen Inc.) per 11 microliter reaction; and 6 ng of genomic DNA. Tubes containing 26, 8.75, 2.9, 0.97, 0.324, and 0.108 ng of a reference DNA (a pooled samples of leukocyte genomic DNA from 100 female donors) are included in each PCR run so that the quantity of targeted templates in each research sample can be determined relative to the reference DNA sample by the standard curve method. The same reference DNA was used for all PCR runs.

To control for inter-assay variability, eight control DNA samples are included in each run. In each batch, the telomere to single copy gene (T/S) ratio of each control DNA is divided by the average T/S for the same DNA from 10 runs to get a normalizing factor. This is done for all eight samples, and the average normalizing factor for all eight samples is used to correct the participant DNA samples to get the final T/S ratio. The T/S ratio for each sample was measured twice. When the duplicate T/S value and the initial value vary by more than 7%, the sample was run the third time and the two closest values were reported. The average coefficient of variation for this study is 1.9%. The lab personnel who performed the assays received de-identified blood samples and were blind to demographic and clinical data.

To determine the conversion factor for the calculation of approximate base pair TL from T/S ratio, the above method was used to determine the T/S ratios, relative to the same reference DNA, for a set of genomic DNA samples from the human fibroblast primary cell line IMR90 at different population doublings, as well as with the telomerase protein subunit gene (hTERT) transfected into a lentiviral construct. The mean TRF length from these DNA samples was determined using Southern blot analysis, and the slope of the plot of mean TRF length versus T/S for these samples served as the conversion factor for calculation of TL in base pairs from the T/S ratio. The equation for conversion from T/S ratio to base pairs for this study was base pairs = 3274 + 2413×(T/S).

### Statistical analysis

SAS version 9.4 (Cary, NC) was used for all analyses. Data were checked for distributional form and outliers. All testing was two sided using a .05 level of significance. Psychological resilience was operationalized based on the sum total of psychological measures indicating resilience (*LOT-R*, *PANAS positive affect*, *PANAS negative affect*, *active coping (CSQ-R)*, and *Perceived Stress Scale (PSS)*). Psychosocial/biobehavioral resilience was operationalized based on the sum total of measures endorsed representing psychological resilience with the addition of three additional measures representing social and biobehavioral factors: *MSPSS*, *tobacco use*, and *WHR*.

Summary statistics were used to describe the sample. Consistent with telomere research recommendations, correlations were computed to assess known (age, sex, and race) and reported (optimism and WHR) associations with TL.^4,9,62^ Regression modeling was used to test Hypotheses 1 and 2. For Hypothesis 1, psychological resilience was entered as a predictor of TL, after controlling for relevant covariates: sex, age, race, and characteristic pain intensity. Similarly, for Hypothesis 2, the psychosocial/biobehavioral resilience composite was entered into a regression model as a predictor of TL, after controlling for relevant covariates: sex, age, race, and characteristic pain intensity. The strength of association between TL and the two resilience measures (i.e., psychological resilience and psychosocial/biobehavioral resilience) was compared using Meng et al.’s^63^ method.

## Results

### Descriptive findings

Of the 79 participants, 67% were females, 58% were NHW with mean age of 58.4 (±8.1) years. A description of the study sample is provided in Table 1. The mean (*SD*) TL was 1.04 (±0.20) with a range from 0.59 to 1.62. Anticipated relationships were demonstrated in that TL was significantly associated with age (*r*_s_ = –31, *p* = .0045) and sex (*r*_s_ = –.25, *p* =.0215) such that females had longer TL, 1.07 (±0.21), than males, 0.97 (±0.18). As previously reported, TL was also significantly associated with optimism, LOT-R (*r*_s_ = .24, *p* =.0341), and WHR (*r*_s_ = –.29, *p* =.0093).^4,9,62^ A description of the reported ranges and sample-specific response patterns for the resilience measures are displayed in Table 2.

**Table 1.** Demographic characteristics of the study sample.

Demographics	% (*N*) or *M* (*SD*)
Age	58.4 (8.1)
Sex	
Female	66% (53)
Male	34% (26)
Ethnicity/race	
Non-Hispanic Black	43% (33)
Non-Hispanic White	57% (46)
Characteristic pain intensity (GCPS)	42.3 (25.9)

GCPS: Graded Chronic Pain Scale.

**Table 2.** Resilience measures and descriptive data.

Domain	Variable	Mean (*SD*)	Range	Indicators	Frequency
Psychological	LOT-R	17 (4.2)	6–24	<18 = 0 ≥18 = 1	41 38
	PANAS-Positive	34.8 (7.1)	15–49	<35 = 0 ≥35 = 1	40 39
	PANSAS-Negative	14.6 (5.4)	10–32	≥18.2 = 0 <18.2 = 1	14 65
	CSQ-R	2.8 (0.8)	0.8–4.7	<2.87 = 0 ≥2.87 = 1	38 41
	PSS	14.1(6.2)	1–34	≥14 = 0 <14 = 1	43 36
Social	MSPSS	64.7 (18.9)	12–84	12–48 = 0 49–84 = 1	12 67
Biobehavioral	Tobacco use	NA	NA	Yes = 0 No = 1	23 55
	WHR	0.9 (0.1)	0.7–1	>0.9 = 0 <0.9 =1	27 52

Note: Lot-R: life orientation test-revised; PANAS: Positive and Negative Affect Scale; CSQ-R: coping strategies questionnaire-revised; PSS: Perceived Stress Scale; MSPSS: Multidimensional Scale of Perceived Social Support; WHR: waist/hip ratio.

### Relationship between psychological resilience and TL

In our regression analysis, we found that the psychological resilience was associated with longer telomeres after controlling for sex, age, race, and characteristic pain (*p* = .030, *b* = .03 (±.02), model *R*^2^ = .21). Table 3 provides the full results for the tested models.

**Table 3.** Psychological and psychosocial/biobehavioral resilience and telomere length.

	Psychological resilience *R*^2^ = 0.21		Psychological resilience with WHR *R*^2^ = 0.23		Psychosocial/biobehavioral resilience *R*^2^ = 0.23	
Variable	*b* (*SE*)	*t*	*b* (*SE*)	*t*	*b* (*SE*)	*t*
Female	0.127 (0.047)	2.73^*^	0.081 (0.309)	1.42	0.102 (.046)	2.19^*^
Non-Hispanic Black	0.037 (0.047)	0.79	0.035 (0.047)	0.73	0.044 (.047)	0.94
Age	–0.008 (0.003)	–3.05^*^	–0.008 (0.003)	–2.63^*^	–0.001 (.003)	–3.11^*^
Characteristic pain intensity (GCPS)	–0.00009 (0.001)	–0.10	0.00001 (0.001)	0.02	–0.0004 (0.001)	–0.06
Psychological resilience composite score	0.035 (0.016)	2.22^*^	0.036 (0.016)	2.27^*^		
WHR			–0.440 (0.315)	–1.40		
Psychosocial/biobehavioralresilience composite score					0.034 (0.013)	2.59^*^

Note: GCPS: Graded Chronic Pain Scale; WHR: waist/hip ratio.

*p < .05.

### Relationship between psychosocial/biobehavioral resilience and TL

The psychosocial/biobehavioral resilience composite was also positively associated with longer telomeres after controlling for sex, age, race, and characteristic pain (*p* = .012, *b* = .03 (±.01), model *R*^2^ = .23). The full model is presented in Table 3.

### Comparison between psychological resilience and psychosocial/biobehavioral resilience

In testing the strength of association between TL and each of the resilience composites, we found that the psychosocial/biobehavioral composite was significantly more associated with TL than the psychological resilience composite (*Z* = –13.23, *p* < .0001).

### Additional analysis

Due to the strong association between WHR and TL, a post hoc analysis was conducted to evaluate the relationship between the psychological resilience composite and TL after adjusting for WHR. The psychological resilience composite remained significantly associated with TL with the addition of WHR as a covariate (*p* = .03, *b* = .04 (±.02), model *R*^2^ = .23). Table 3 provides the full results for the tested model.

Additionally, to further assist with the interpretation of the clinical relevance, an analysis of covariance was completed comparing individuals with low levels of psychosocial/biobehavioral resilience (sum values in the 1–3 range) compared to those individuals with high levels of psychosocial/biobehavioral resilience (values in the 5–8 range). The T/S ratios for both groups were converted to base pairs as described in the Methods section. The groups differed by 256 base pairs. Applying findings from a prior publication, a difference of 256 base pairs suggests a general indication of an approximate 10 years difference in cellular aging.^64^ As base pairs were not directly measured in the current cohort, and the conversion formula is based on a series of DNA samples from a human primary cell line, not a direct comparison of the Southern blot method and quantitative PCR, interpretation is limited and can only provide a frame of reference for evaluating possible clinical relevance.

## Discussion

Our findings provide evidence that in individuals with and without knee pain, higher levels of both psychological and psychosocial/biobehavioral resilience were associated with longer telomeres. Importantly, the broader conceptualization of resilience including psychosocial and biobehavioral factors provided the strongest association with TL compared to psychological resilience alone. Thus, a resilience phenotype characterized by a greater array of psychosocial and biobehavioral protective factors is associated with longer telomeres.

### Psychological resilience and TL

Psychological resilience has frequently been associated with enhanced emotional, cognitive, social, and physical functioning. Numerous publications report that positive psychological factors buffer the experience of chronic pain as indicated by lower report of clinical symptoms and functional limitations.^17–20,65^ Additionally, there is a growing body of evidence linking psychological resilience factors with biological benefits.^66,67^ A relationship between longer telomeres and positive psychological factors, particularly optimism, has been shown.^9^ In this study, we also found a correlation between optimism and TL. Our research contributes to the body of evidence indicating that psychological resilience factors have a biological interface.

### Psychosocial/biobehavioral resilience and TL

A number of publications have addressed the role of social support in chronic pain, with most describing its benefits and a few noting that some forms of social support (e.g., the reinforcement of maladaptive behavior patterns) may be detrimental in chronic pain.^68,69^ However, the health benefits of social support have been consistently indicated,^24–27^ including associations with improved stress-related physiological functioning.^28,29^ Also, negative social experiences (e.g., social isolation and loneliness) are associated with multiple health risks, including inactivity, smoking, high blood pressure, and shorter telomeres in individual who also demonstrated dysregulated parasympathetic functioning.^70–73^

As expected, health behaviors, e.g., regular exercise, maintaining a healthy weight, healthy diet, moderate alcohol consumption, and not smoking, are also associated with lower levels of disease, biological burden, and death across a range of studies including those specific to individuals with chronic pain.^35–37,74^ Telomeres have been associated with an array of biopsychosocial positive factors, our findings further contribute to this body evidence.^7,67,75,76^ In our study, a linear relationship emerged such that an increase of psychosocial/biobehavioral measures representing resilience was significantly associated with longer telomeres.

### Additional considerations

Telomeres can be conceptualized as a downstream measure of persisting overall stress system functioning. As such, a limited association between TL and specific psychological, social, and biobehavioral measures would be expected would not typically reach a “dose level” to contribute in a meaningful way to TL.^8,77^ However, combined, these factors characterize a resilience phenotype which could represent traits and behaviors of sufficient magnitude to buffer against telomere shortening.^67,77^ Our findings suggest that there is a positive linear relationship between TL and resilience characteristics in individuals with or at risk for knee OA. Additionally, we previously reported a dose-response relationship between chronic pain severity and TL, such that differences in TL between those with the highest chronic pain severity compared to individuals with no or low levels of chronic pain severity was 16 years of accelerated aging.^16^ Similarly, in the current study, based on conversion estimations, individuals with the lowest psychosocial/biobehavioral resilience scores compared to those with the highest psychosocial/biobehavioral resilience scores differed by approximately 10 years of cellular aging even after controlling for relevant covariates.

### Limitations and future directions

There are a number of limitations warranting acknowledgment. As this investigation is a cross-sectional study with a relatively small sample, prospective investigations with a larger sample are necessary. Additionally, our sample is comprised of middle-aged and older adults, many who screened positive for or are at risk for knee OA, thus the generalizability is limited. Future investigations with individuals with differing chronic pain conditions will be necessary. Our resilience measures were selected from those available in a completed study. There are a number of other factors and measures that warrant evaluation in better understanding the biological interface of resilience in chronic pain conditions. Finally, telomere research is still a developing science with recognized limitations.^77^ Strategies to increase confidence were incorporated in the current study by working with a recognized and well-published lab, Blackburn Lab, University of California San Francisco, and evaluating known patterns between TL with age, sex, and ethnicity/race.

There are a number of additional strengths that are noteworthy in the current investigation. The UPLOAD study provides a well-characterized sample with a broad array of measures to answer the identified questions. The resilience measures were comprised of validated instruments and measures with recognized norms to define ranges for resilience. Characterizing a phenotype based on a combination of measures provides a stronger representation than any one measure alone.^77^ In regard to future directions, there are significant disparities in the clinical and functional limitations of knee OA in NHB and NHW. Investigations exploring potential race and ethnic group differences in resilience factors might highlight factors contributing to those disparities and illuminate possible targets for treatment. Lastly, if findings are replicated, the clinical implications are exciting. There is a strong body of evidence indicating resilience factors (psychological traits and health behaviors) are associated with lower rates of morbidity and mortality. Our findings suggest the biological interface of resilience factors is measurable. Hence, clinical strategies to increase resilience and improve pain-related health outcomes may also not only buffer the biological burden of pain but findings suggest that we may be able to monitor and evaluate the biological benefits of various interventions.

## Conclusions

In summary, our findings demonstrate a positive relationship between resilience factors and TL. Specifically, in middle-aged and older adults with or at risk for knee OA, greater psychosocial and biobehavioral resilience was significantly associated with longer telomeres. Further, we have previously shown a dose-response relationship between TL and increasing chronic pain severity.^16^ Current findings suggest that there is also a dose-response relationship between psychosocial/biobehavioral resilience factors and TL. Second, our findings support prior recommendations of the benefits of considering combining measures in better capturing the biological interface to particular phenotypes, in this study, resilience phenotypes.^67,77^ Third, our study has potential clinical relevance. If findings from the current study are replicated and extended into prospective analyses which indicate an influence on health outcomes, then psychosocial and biobehavioral resilience interventions and measures could be used to guide and evaluate treatment for chronic pain.

## Authors' Note

Toni L Glover is now affiliated with School of Nursing, Oakland University, Rochester, MI, USA.
